# Atopic Dermatitis and Probiotic Use in Children Aged 1–4 Years: Findings from a Nationwide Survey in Serbia

**DOI:** 10.3390/medsci14060647

**Published:** 2026-10-08

**Authors:** Maja Đanić, Nikolija Marković, Tijana Ostojić, Bojan Stanimirov, Borko Milanović, Saša Vukmirović, Nebojša Pavlović

**Affiliations:** 1 Department of Pharmacology, Toxicology and Clinical Pharmacology, Faculty of Medicine, University of Novi Sad, 21000 Novi Sad, Serbia; sasa.vukmirovic@mf.uns.ac.rs; 2 Faculty of Medicine, University of Novi Sad, 21000 Novi Sad, Serbia; 014921@mf.uns.ac.rs; 3 Department of Fundamental Sciences, Faculty of Technical Sciences, University of Novi Sad, 21000 Novi Sad, Serbia; tijana.ostojic@uns.ac.rs; 4 Department of Biochemistry, Faculty of Medicine, University of Novi Sad, 21000 Novi Sad, Serbia; bojan.stanimirov@mf.uns.ac.rs; 5 Institute for Child and Youth Healthcare of Vojvodina, 21000 Novi Sad, Serbia; borko.milanovic@mf.uns.ac.rs; 6 Department of Pediatrics, Faculty of Medicine, University of Novi Sad, 21000 Novi Sad, Serbia; 7 Department of Pharmacy, Faculty of Medicine, University of Novi Sad, 21000 Novi Sad, Serbia; nebojsa.pavlovic@mf.uns.ac.rs

**Keywords:** atopic dermatitis, probiotics, children, parental attitudes, gut microbiota, gut–skin axis, early-life factors, immune modulation

## Abstract

Background/Objectives: The potential role of probiotics in the prevention and management of atopic dermatitis (AD) has received increasing attention. This study aimed to investigate factors associated with parent-reported AD in children aged 1–4 years in Serbia and to characterize patterns of probiotic use and parental attitudes toward probiotics. Methods: A nationwide cross-sectional online survey was conducted among parents of 1153 children aged 1–4 years in Serbia. Associations between AD and demographic, perinatal, feeding, and probiotic-related characteristics were assessed using multivariable logistic regression. Parental attitudes toward probiotics were evaluated using a three-item Likert-scale instrument, and patterns of probiotic use were analyzed among children with AD. Results: Parent-reported AD was present in 23.6% of children. Cesarean delivery (aOR = 1.562; 95% CI: 1.179–2.069), post-term birth (aOR = 1.862; 95% CI: 1.139–3.044), and probiotic use during the previous 12 months (aOR = 1.645; 95% CI: 1.214–2.231) were independently associated with higher odds of parent-reported AD in children. Previous probiotic use was highly prevalent among children with AD, although only 35.8% were reported to use probiotics for skin-related purposes. Parents of children with AD had slightly more favorable attitudes toward probiotics, primarily regarding their potential role in preventing AD. Conclusions: These findings highlight the complex relationship between early-life factors, probiotic use, and AD and underscore the importance of evidence-based parental information and guidance from healthcare professionals regarding probiotic use in pediatric AD. Because of the cross-sectional design, these associations should not be interpreted as evidence of a causal relationship between probiotic use and AD.

## 1. Introduction

Atopic dermatitis (AD) is one of the most common chronic inflammatory skin diseases of childhood, characterized by recurrent episodes of eczema, intense pruritus, xerosis, and impaired skin barrier function. The global prevalence of AD has increased considerably over recent decades, making it one of the most common chronic diseases in childhood and an important public health concern [1]. The development of AD is multifactorial, involving genetic susceptibility, environmental factors, immune dysregulation, and alterations in skin barrier integrity [2]. The burden of AD extends beyond dermatological symptoms. Children with AD frequently experience sleep disturbances, emotional distress, anxiety, and impaired social functioning, while parents often report increased caregiving burden and reduced quality of life. The bidirectional interaction between psychological stress and immune dysfunction may further exacerbate disease activity, emphasizing the need for comprehensive disease management that addresses both biological and psychosocial aspects of AD [3].

Growing evidence indicates that alterations in both gut and skin microbiota contribute to the pathogenesis and progression of AD. The microbiota play essential roles in maintaining immune homeostasis, epithelial barrier integrity, and host defense against pathogens. These microbial communities interact continuously with host tissues and immune cells, contributing to the regulation of inflammatory responses and helping to maintain the physiological integrity of the intestinal and cutaneous barriers [4]. A balanced intestinal microbiome promotes immune tolerance by regulating T helper cell responses and inducing regulatory T cells, whereas microbial dysbiosis has been associated with chronic inflammation and allergic diseases. Such alterations in microbial composition may disrupt the balance between pro-inflammatory and regulatory immune pathways, potentially contributing to an exaggerated immune response in susceptible individuals [5]. The concept of the gut–skin axis has therefore emerged as an important mechanism linking intestinal microbial composition with skin health. Several studies have demonstrated reduced gut microbial diversity in children with AD, characterized by lower abundances of beneficial genera such as *Lactobacillus* and *Bifidobacterium* and increased levels of potentially pathogenic microorganisms. These alterations appear particularly important during early childhood, when the gut microbiota undergoes rapid maturation and plays a critical role in immune system development. During this period, environmental and perinatal factors can substantially influence microbial colonization, potentially affecting the establishment of immune tolerance and the subsequent risk of allergic disease [6,7].

The growing understanding of the gut–skin axis has led to increasing interest in probiotics as a potential adjunctive strategy for the prevention and management of pediatric AD. Their potential role is particularly relevant in early childhood, when the intestinal microbiota and immune system are still developing and may be more responsive to environmental and dietary influences. Probiotics are defined as live microorganisms that confer health benefits on the host when administered in adequate amounts. Their proposed mechanisms include modulation of gut microbial composition, enhancement of intestinal barrier function, regulation of immune responses, and promotion of immune tolerance [6]. By interacting with the intestinal microbiota and host immune system, probiotics may help restore microbial balance and influence inflammatory pathways that are relevant to the pathogenesis of AD. In particular, strains belonging to the *Lactobacillus* and *Bifidobacterium* genera have been most extensively investigated because of their immunomodulatory properties and their reduced abundance in children with AD [8]. However, probiotic effects are considered to be highly strain-specific, meaning that findings observed with one probiotic strain or combination cannot necessarily be extrapolated to other products. This strain-specificity is an important consideration when interpreting clinical studies and when translating available evidence into clinical practice. Although randomized clinical trials and recent meta-analyses suggest that probiotic supplementation may modestly reduce disease severity, particularly as measured by SCORAD, the available evidence remains heterogeneous owing to differences in probiotic strains, treatment duration, patient populations, and study design [3,9].

Because probiotic products are widely available without prescription, parents play a central role in decisions regarding their use during early childhood. Their attitudes, beliefs, and expectations play an important role in determining whether probiotics are used appropriately, particularly in children with chronic conditions such as AD. Misconceptions regarding probiotic efficacy, safety, strain specificity, or indications may contribute to inappropriate use or unrealistic treatment expectations. Parents may also perceive probiotics as universally beneficial or interchangeable between different products, despite the fact that probiotic effects are highly strain-specific and may vary according to the indication and characteristics of the child. In addition, the increasing availability of probiotic products through pharmacies, supermarkets, and online sources provides parents with access to a wide range of products, which may make informed selection more challenging. Therefore, access to reliable and evidence-based information is essential for supporting appropriate probiotic use, particularly in young children with chronic inflammatory conditions such as AD. Healthcare professionals can play an important role in this process by providing individualized recommendations, explaining the current evidence, and helping parents develop realistic expectations regarding the potential benefits and limitations of probiotic supplementation [3,10].

In addition to microbial and immunological factors, several perinatal and early-life characteristics have been investigated as potential determinants of AD development. Factors such as mode of delivery, breastfeeding, antibiotic exposure, and other early-life exposures may influence susceptibility to AD, although findings across studies remain inconsistent. These factors may affect the early development of the immune system and the establishment of the infant microbiome, potentially influencing the risk of subsequent allergic disease. Therefore, identifying modifiable early-life factors may be important for better understanding the development of AD and for informing potential preventive strategies [11].

Despite increasing interest in probiotics and the gut–skin axis, data integrating probiotic use, parental attitudes, and factors associated with AD in young children remain limited, particularly in Central and Eastern European countries.

To address this knowledge gap, the present study aimed to determine the prevalence of parent-reported AD and to examine its association with selected perinatal and early-life characteristics and probiotic use among children aged 1–4 years in Serbia using data from a nationwide cross-sectional survey. In addition, the study characterized parental attitudes toward probiotics and patterns of previous probiotic use among children with AD.

## 2. Materials and Methods

### 2.1. Study Design and Participants

This cross-sectional study was based on data collected through a nationwide online survey conducted in Serbia between November 2024 and January 2025. The survey was designed to investigate parental knowledge, attitudes, and practices regarding probiotic use in children.

The present study represents a secondary analysis of the survey dataset and addresses a distinct research objective by examining previously unpublished questionnaire items related to AD together with selected variables on probiotic use and parental attitudes in young children. The survey methodology has been described previously [12].

Parents or legal guardians of children aged 1–4 years were eligible for inclusion in the present analysis. The questionnaire was administered electronically using Google Forms and disseminated through informal social media groups targeting parents in Serbia. Recruitment used a voluntary convenience-sampling approach. Participation was anonymous and voluntary, and electronic informed consent was obtained from all participants before accessing the questionnaire. Only fully completed questionnaires were included in the analysis. Because the survey invitation was disseminated through social media groups and the number of individuals who were exposed to the invitation could not be reliably determined, a conventional response rate could not be calculated.

The study was approved by the Ethics Committee of the Faculty of Medicine, University of Novi Sad (Approval No. 01-39/278; Approval date: 29 November 2024) and conducted in accordance with the principles of the Declaration of Helsinki.

### 2.2. Questionnaire

The questionnaire consisted of three sections covering parental and child demographic characteristics, pregnancy and birth characteristics, early-life feeding practices, child health status, probiotic use, and parental attitudes toward probiotic supplementation.

The first section collected demographic information, including parental age, place of residence, education, occupation, and healthcare profession, together with child-related characteristics such as sex, age, mode of delivery, gestational age at birth, infant feeding practices, dietary habits, and general health status.

The second section assessed parent-reported medical history during the previous 12 months, including medication use and history of physician-diagnosed AD. Information regarding probiotic administration, duration of use, probiotic composition, indications for supplementation, and factors influencing probiotic selection was also collected. Although detailed information on probiotic indication, duration of use, and reported composition was collected, probiotic exposure in the primary multivariable analysis was operationalized as a binary variable indicating any probiotic use during the previous 12 months.

The final section assessed parental attitudes toward probiotics using Likert-scale statements evaluating beliefs regarding long-term probiotic use, immune benefits, and the potential role of probiotics in preventing AD and other allergic skin diseases.

Two positively worded statements were scored from 1 (strongly disagree) to 5 (strongly agree). The negatively worded statement was reverse-scored from 1 (strongly agree) to 5 (strongly disagree). The three item scores were summed producing a total parental attitude score ranging from 3 to 15 with higher scores indicating more favorable attitudes toward probiotic use.

### 2.3. Statistical Analysis

Following data collection, questionnaire responses were exported in CSV format, imported into Microsoft Excel for data management, and subsequently analyzed. Statistical analyses were performed using IBM SPSS Statistics (version 22.0, SPSS Inc., Chicago, IL, USA). Categorical variables were summarized using absolute and relative frequencies (*n* and %). Associations between categorical variables and AD were assessed using Pearson’s χ^2^ test.

The prevalence of parent-reported AD in children was calculated for each category of the analyzed variables. Variables considered clinically relevant or showing evidence of association in the bivariate analysis were entered into a multivariable logistic regression model to identify independent factors associated with AD. The results of the logistic regression analysis were presented as odds ratios (OR) with corresponding 95% confidence intervals (95% CI). The following variables were included in the model: child sex, age, mode of delivery, gestational age at birth, type of infant feeding, and probiotic use. Reference categories were predefined (female sex, age of 4 years, vaginal delivery, term birth, exclusive breastfeeding, and no probiotic use).

Additionally, to assess potential effect modification, an interaction term between probiotic use and mode of delivery was introduced into the model. This interaction term was created as the product of these two variables to evaluate whether the association between probiotic use and AD differed according to the mode of delivery. Model fit was evaluated using the Hosmer–Lemeshow goodness-of-fit test, while the proportion of explained variance was assessed using the Nagelkerke R^2^ coefficient. A *p*-value < 0.05 was considered statistically significant.

The total parental attitude score was compared between parents of children with and without AD using the Mann–Whitney U test. For individual attitude items, differences in the distribution of responses between the two groups were assessed using Pearson’s χ^2^ test. To account for multiple testing, *p*-values for the three item-level comparisons were adjusted using the Holm method. The strength of associations was assessed using Cramer’s V. A two-sided *p*-value < 0.05 was considered statistically significant.

## 3. Results

Detailed demographic and clinical characteristics of the study population are presented in Table 1. A total of 1153 parents of children aged 1–4 years were included in the analysis. The study population comprised 614 (53.3%) girls and 539 (46.7%) boys. The age distribution was relatively balanced across all four age groups, with the largest proportion of children being 1 year old (27.2%). Most children were born by vaginal delivery (62.1%) and at full term (88.0%), while 55.4% were exclusively breastfed during the first months of life. More than half of the participants (52.3%) came from single-child families. Overall, AD was reported in 23.6% of children while 64.8% of parents-reported probiotic use in their children during the previous 12 months.

Association between demographic and early-life characteristics and the presence of AD is presented in Table 2. No significant differences were observed according to child sex, age, number of children in the family, or infant feeding practices (all *p* > 0.05). AD was more frequently reported among children born by cesarean section than among those born vaginally (28.6% vs. 20.5%, *p* = 0.002). In addition, AD was more frequently reported among children who had received probiotics during the previous 12 months compared with those who had not (26.6% vs. 18.0%, *p* = 0.001). Gestational age at birth showed a borderline association with AD (*p* = 0.052), with the highest prevalence observed among post-term children (34.6%).

Mode of delivery, gestational age at birth, and probiotic use were significantly associated with AD. Children born by cesarean section had 56% higher odds of AD compared with those born vaginally (aOR = 1.562, 95% CI: 1.179–2.069, *p* = 0.002). The post-term group comprised 81 children, and post-term birth was associated with higher odds of parent-reported AD compared with full-term birth (aOR = 1.862, 95% CI: 1.139–3.044, *p* = 0.013), whereas preterm birth was not significantly associated with the outcome (aOR = 1.003, 95% CI: 0.531–1.892, *p* = 0.993). Previous 12-month probiotic use was independently associated with higher odds of parent-reported AD (aOR = 1.645, 95% CI: 1.214–2.231, *p* = 0.001). In contrast, child sex, child age, and infant feeding type were not significantly associated with AD in the adjusted model (all *p* > 0.05) (Table 3).

The multivariable logistic regression model was statistically significant (χ^2^(10) = 30.630, *p* = 0.001), with a Nagelkerke R^2^ of 0.039 and a non-significant Hosmer–Lemeshow test (χ^2^(8) = 15.229, *p* = 0.055). Although the overall model was statistically significant, its explanatory power was limited, indicating that the included variables accounted for only a small proportion of the variability in AD status.

To investigate whether the association between probiotic use and AD differed according to the mode of delivery, an interaction term between probiotic use and mode of delivery was included in the multivariable logistic regression model. No statistically significant interaction between probiotic use and mode of delivery was identified in the multivariable logistic regression model (*p* = 0.630), suggesting that the association between probiotic use and AD was similar among children born vaginally and those delivered by cesarean section.

The mean parental attitude score among all respondents was 10.64 ± 2.23 while the median score was 11 (IQR: 9–12) on a scale ranging from 3 to 15. Parents of children with AD had a significantly higher median score than parents of children without AD: 11 (IQR: 10–13) versus 10 (IQR: 9–12), respectively (Mann–Whitney U = 99,727, Z = −4.224, *p* < 0.001, r = 0.124). Despite statistical significance, the magnitude of the difference was small.

**Figure 1:**
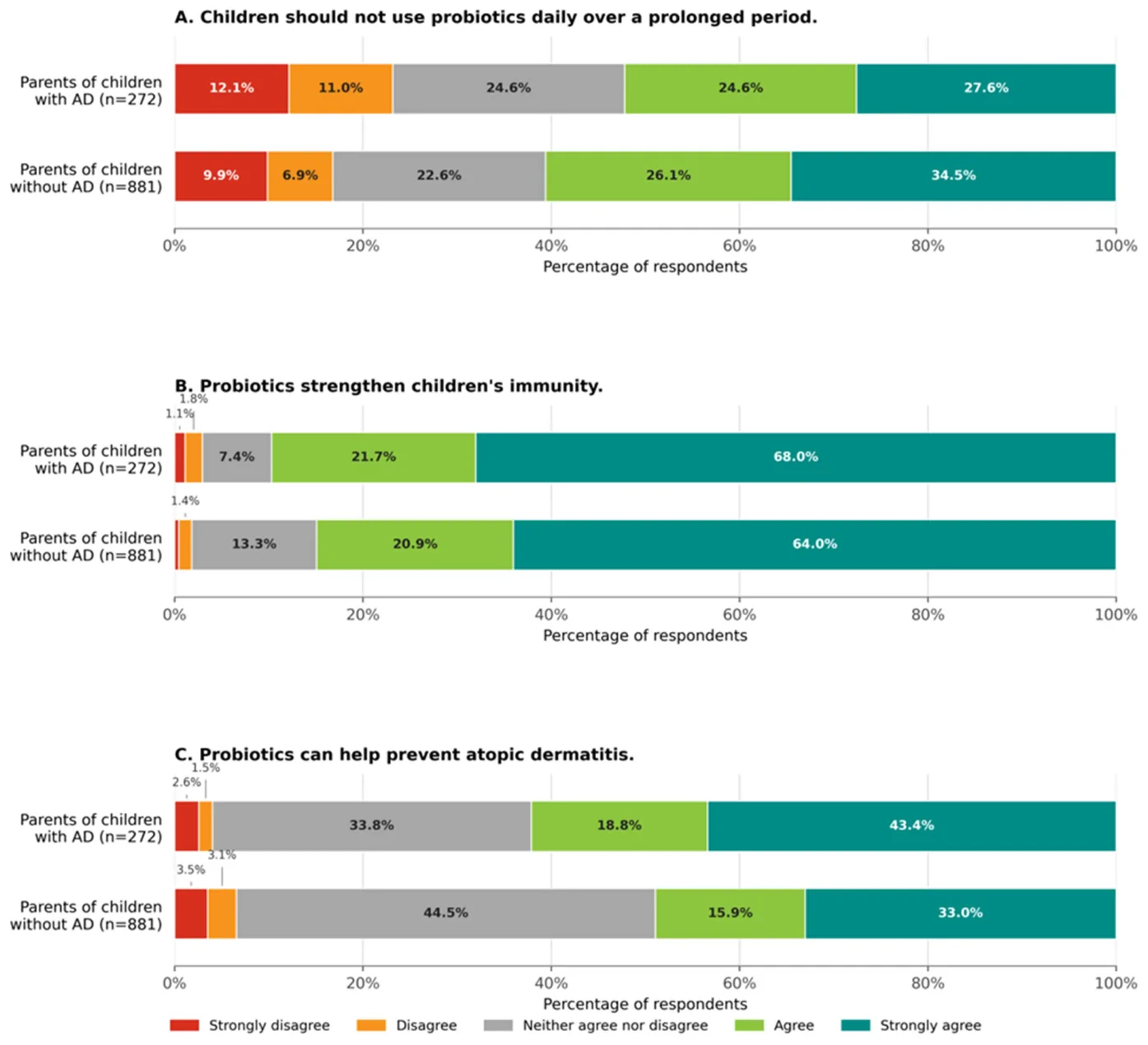
Distribution of parental attitudes toward probiotic use according to the child’s AD status. Bars represent percentages calculated within each AD group. The negatively worded statement in panel (**A**) was reverse-scored when calculating the total parental attitude score. AD-atopic dermatitis.

To further characterize the attitudes underlying the overall score, responses to the three individual statements were compared between the groups (Figure 1). The distribution of responses did not differ significantly between parents of children with and without AD for the statement concerning prolonged daily probiotic use (χ^2^(4) = 9.047, *p* = 0.060, Cramer’s V = 0.089, Holm-adjusted *p* = 0.120) or for the statement that probiotics strengthen children’s immunity (χ^2^(4) = 8.482, *p* = 0.075, Cramer’s V = 0.086, Holm-adjusted *p* = 0.120). In contrast, responses to the statement that probiotics can help prevent AD differed significantly between the groups (χ^2^(4) = 15.470, *p* = 0.004, Cramer’s V = 0.116; Holm-adjusted *p* = 0.012). Parents of children with AD more frequently strongly agreed with this statement than parents of children without AD (43.4% vs. 33.0%). The magnitude of this association was small.

Among the 272 children with AD, 265 (97.4%) had received probiotics at some point in the past. Among children with a history of probiotic use, the most frequently reported reasons were use during antibiotic therapy (69.4%) and for gastrointestinal complaints (65.3%). Probiotics were also used for the prevention or reduction of skin symptoms in 35.8% of children. Physician recommendation was the most frequently reported factor influencing probiotic selection (62.6%), followed by pharmacist recommendation (38.9%). Lactobacillus-containing products were the most frequently reported probiotic composition (34.3%), while 25.7% of children had received probiotics for more than 6 months (Table 4).

## 4. Discussion

This nationwide cross-sectional study investigated factors associated with AD among 1153 children aged 1–4 years, with a particular focus on probiotic use and selected perinatal characteristics. The prevalence of parent-reported AD in the study population was 23.6%, which is consistent with previously reported rates in early childhood populations across Europe, where prevalence typically ranges between 15% and 30%, depending on diagnostic criteria and age groups [13].

The principal findings of the present study were that cesarean delivery, post-term birth, and probiotic use were independently associated with higher odds of AD in the multivariable model. In contrast, no significant associations were observed for child sex, age, infant feeding type, or number of children in the family in the bivariate analyses, while child sex, age, and infant feeding type were not significantly associated with AD in the adjusted model.

The prevalence of parent-reported AD was higher among children who had received probiotics during the previous 12 months than among those who had not (26.6% vs. 18.0%). This association remained statistically significant after adjustment for child sex, age, mode of delivery, gestational age and infant feeding type, with probiotic use associated with higher odds of parent-reported AD. However, this finding should not be interpreted as evidence that probiotic use increases the risk of AD, given the cross-sectional design of the study. In particular, reverse causality and indication bias cannot be excluded, as parents of children with AD or other health problems may have been more likely to administer probiotics. The high prevalence of previous probiotic use among children with AD in our study further supports the possibility that probiotic administration may have been influenced by the child’s health status or parental perceptions regarding the potential benefits of probiotics. The observed association therefore most likely reflects a complex relationship between probiotic use, underlying health conditions, and parental treatment-seeking behavior rather than a direct effect of probiotics on the development of AD. Nevertheless, this finding is relevant from a real-world perspective, as it demonstrates that probiotic use is particularly common among young children with AD. A comparable pattern has recently been reported in the population-based Northern Finland Birth Cohort, in which regular probiotic use was more frequent among adults with self-reported physician-diagnosed AD than among those without AD (16.3% vs. 11.3%) [14]. Although the population and age group are different from those in our study, this finding supports the possibility that individuals affected by AD are more likely to seek and use dietary supplements, rather than indicating that probiotic exposure contributes to the development of AD.

Several randomized controlled trials and meta-analyses have reported modest reductions in disease severity, most commonly reflected by improvements in SCORAD scores following probiotic supplementation; however, the overall evidence remains inconsistent and the clinical relevance of these effects remains uncertain [15,16]. The biological plausibility of a relationship between probiotics and AD is further supported by evidence that the gut microbiota participates in immune maturation and regulation of allergic inflammation. Probiotic microorganisms may influence these processes through immunomodulatory mechanisms, although the extent to which these effects translate into clinically meaningful improvements in AD remains uncertain [17].

Children born by cesarean section showed a higher prevalence of parent-reported AD compared to those born vaginally (28.6% vs. 20.5%). After adjustment for other perinatal and early-life characteristics, cesarean delivery remained independently associated with higher odds of parent-reported AD. Children born by cesarean section had 56% higher odds of AD than those born vaginally.

This finding is biologically plausible, as cesarean delivery may interfere with the establishment of the neonatal gut microbiota and early immune maturation by limiting exposure to the maternal vaginal and intestinal microbiota [18,19]. Altered microbial colonization during this critical developmental period has been proposed as one of the mechanisms contributing to an increased susceptibility to allergic diseases, including AD [20,21]. However, findings from large epidemiological studies remain inconsistent. A recent population-based cohort study conducted within the Kaiser Permanente Northern California healthcare system, including over 173,000 children, reported only a weak unadjusted association between cesarean delivery and AD (RR = 1.06; 95% CI: 1.03–1.10), which was attenuated to the null after adjustment for confounding factors (aRR = 1.02; 95% CI: 0.99–1.05) [22]. Furthermore, analyses accounting for intrapartum antibiotics, breastfeeding, and familial factors did not demonstrate a meaningful independent association between cesarean delivery and AD in that population [22]. This discrepancy may reflect differences in study design, sample size, population characteristics, exposure and outcome assessment, and the range of confounding factors available for adjustment. Residual confounding may also be relevant, particularly because our survey did not capture all potentially important familial, environmental, or perinatal factors. Overall, although a biological link between cesarean delivery, early microbial colonization, and immune development remains plausible, the present findings should be interpreted as an association rather than evidence of a causal effect of cesarean delivery on AD [22].

Given the altered initial microbial colonization observed in infants delivered by cesarean section, it could be hypothesized that the association between probiotic use and AD might differ according to the mode of delivery. However, no statistically significant interaction between probiotic use and mode of delivery was observed in the present study, suggesting that the association between probiotic use and AD was similar regardless of whether the child was born vaginally or by cesarean section.

Gestational age at birth was significantly associated with AD in the multivariable model. Compared with full-term birth, post-term birth was associated with higher odds of parent-reported AD, whereas no association was observed for preterm birth. The post-term group in our study was substantially smaller than the full-term group; however, which may have resulted in less precise estimates and greater susceptibility to random variation and residual confounding. Therefore, the observed association should be interpreted cautiously and does not establish post-term birth as a causal risk factor for AD. Previous studies have reported inconsistent findings regarding the association between gestational age and AD. In a large nationwide population-based study from Korea, preterm birth was associated with a lower risk of AD compared with full-term birth [23]. This finding differs from our results, as we found no significant association between preterm birth and AD. Although preterm birth has been linked to immune immaturity and altered early microbial colonization, the extent to which these differences influence subsequent allergic disease remains unclear [24]. Differences in study populations, study design, definitions of gestational-age categories and AD, and the number of preterm children may partly explain the discrepant findings. In contrast, the association observed for post-term birth in our study was consistent with previous population-based evidence. In a large nationwide Finnish study including 965,203 children, post-term birth (≥42 weeks of gestation) was associated with a slightly increased risk of AD compared with full-term birth (adjusted OR 1.06, 95% CI 1.01–1.10) [25]. Although the strength of the association in our study was greater, the direction of the association was similar. Larger prospective studies with more balanced representation across gestational-age categories are needed to determine whether this association is reproducible and to clarify its potential mechanisms.

No significant association was observed between infant feeding type and AD in either the unadjusted analysis or the multivariable model. Compared with exclusive breastfeeding, neither mixed feeding nor exclusive formula feeding was significantly associated with parent-reported AD. Our findings are consistent with some previous evidence suggesting that breastfeeding may not be independently associated with the risk of AD. A systematic review and meta-analysis of 27 prospective cohort studies found no significant association between exclusive breastfeeding and AD (pooled RR = 0.99; 95% CI: 0.88–1.11), although substantial heterogeneity was observed across the included studies [26]. However, evidence from individual studies has suggested a potentially protective effect of exclusive breastfeeding against allergic diseases. For example, a cross-sectional study conducted in Sudan reported a lower prevalence of atopic diseases, including eczema, among exclusively breastfed children compared with children who were not exclusively breastfed during the first six months of life [27]. Differences in study design, population characteristics, duration and exclusivity of breastfeeding, as well as definitions of both feeding exposure and atopic outcomes, may contribute to discrepancies between studies. In the present study, infant feeding was classified according to the reported feeding type rather than detailed duration or timing of breastfeeding and complementary feeding. This relatively broad categorization may have limited our ability to detect associations related to specific breastfeeding patterns or duration of exclusive breastfeeding. Therefore, the lack of a significant association in our study should be interpreted in the context of how infant feeding was assessed and the heterogeneous evidence reported in the literature, rather than as evidence that breastfeeding has no influence on the development of AD.

An additional finding of the present study was the difference in parental attitudes toward probiotic use according to the child’s AD status. Parents of children with AD had a slightly higher median overall attitude score than parents of children without AD. This suggests that the presence of AD may be associated with somewhat more favorable parental perceptions of probiotic use. However, the small effect size indicates that the practical difference in overall attitudes between the groups was limited.

Analysis of the individual attitude statements provided a more specific interpretation of this finding. After adjustment for multiple comparisons using the Holm method, only responses to the statement that probiotics can help prevent AD differed significantly between the groups. Parents of children with AD were more likely to strongly agree with this statement than parents of children without AD (43.4% vs. 33.0%). This pattern may reflect greater parental exposure to information, recommendations, or personal experiences related to probiotic use in the context of their child’s condition. In contrast, responses concerning prolonged daily probiotic use and the potential of probiotics to strengthen children’s immunity did not differ significantly between the groups after adjustment. Thus, the difference in attitudes was primarily related to the perceived preventive role of probiotics in AD rather than to a generally more favorable view of probiotics. This finding differs from a qualitative study among Danish parents of young children, in which parents were generally willing to use probiotics when their child was ill but expressed greater skepticism toward their preventive use. In that study, probiotics were more often perceived as a treatment than as a preventive intervention [28]. The different patterns may reflect differences in the clinical context and in parents’ perceptions of the intended role of probiotics.

The pattern of probiotic use among children with AD provides an important insight into the reasons why these products are used in routine practice. Although probiotic use was almost universal among children with AD in our study, their use specifically for skin-related symptoms was reported by only 35.8% of parents. Probiotics were more commonly administered during antibiotic therapy or in response to gastrointestinal complaints, indicating that their use among children with AD was predominantly related to other common pediatric indications rather than to the management or prevention of AD itself. This distinction is important when interpreting the observed association between probiotic use and AD, as probiotic exposure in this population does not necessarily represent an intervention directed at the skin disease.

This pattern is consistent with previous research on probiotic use in young children. In a cross-sectional survey of parents of preschool children, gastrointestinal complaints and antibiotic use were among the most frequently reported reasons for probiotic administration [29]. Taken together, these findings suggest that probiotics are widely incorporated into pediatric care for several common indications, while their use specifically in the context of AD remains relatively limited.

A somewhat different pattern was observed in a small Polish online survey of 70 adults with AD who had used probiotics for at least five days. In that study, 56% reported previous probiotic use specifically to alleviate AD symptoms, while 62% expressed satisfaction with probiotic therapy, most commonly because of a perceived reduction in allergic symptoms [30]. However, direct comparison with our findings is limited by the adult and highly selected study population, and reliance on retrospectively self-reported outcomes without a control group. Nevertheless, these results suggest that skin-directed probiotic use and perceived benefit may be more common among individuals already engaged with probiotics as a complementary approach to AD management.

*Lactobacillus*-containing preparations were the most frequently reported (34.3%), followed by combinations of strains (26.4%), while 27.9% of parents did not consider or know the probiotic composition. This is clinically relevant because probiotic effects are strain- and formulation-specific and cannot be inferred from genus-level composition alone. Duration of use also varied considerably: 45.7% of children had received probiotics for no longer than 30 days, whereas 25.7% had used them for more than six months. Some clinical studies and subgroup analyses suggest that supplementation for at least eight weeks may be more likely to produce detectable improvement; however, findings regarding the optimal duration remain inconsistent, and no universal minimum treatment period can currently be defined [16,31]. Interpretation is further limited because duration in our survey represented the longest period of probiotic use for any indication rather than the duration of an AD-specific treatment course.

Healthcare professionals were also important sources of product selection, with 62.6% of parents reporting physician recommendations and 38.9% pharmacist recommendations. Considering their important role in product selection, their ability to provide indication- and strain-specific advice is particularly important. In a survey of dermatologists and family physicians in Saudi Arabia, only 23.1% demonstrated good overall knowledge regarding probiotics in AD, while 66.7% identified insufficient knowledge as a barrier to their use in practice [32]. Although obtained in a different healthcare setting, these results emphasize that professional recommendations should be supported by accessible and up-to-date evidence on specific probiotic formulations and realistic treatment expectations.

Overall, these findings indicate that probiotic use among children with AD is frequent and heterogeneous, with substantial variation in indication, duration, and product composition, while healthcare professionals appear to play an important role in product selection.

This study has several limitations that should be considered when interpreting the findings. First, its cross-sectional design precludes causal inference and does not allow conclusions regarding the temporal relationship between probiotic use and AD. Consequently, reverse causality and indication bias cannot be excluded. Second, participants were recruited through voluntary online convenience sampling via social media, which may have introduced selection bias. Parents with greater interest in child health, probiotics, allergic diseases, or related topics may have been more likely to participate. This recruitment strategy may therefore limit the representativeness of the sample and the generalizability of the findings to the broader population of parents of children aged 1–4 years in Serbia. Third, AD status was based on parental reporting of a previous physician diagnosis and was not independently verified through medical records or clinical examination. Furthermore, disease severity was not assessed using validated clinical instruments such as SCORAD, precluding evaluation of whether probiotic use differed according to AD severity. Finally, although information on probiotic composition, indication, and duration of use was collected, these characteristics were not incorporated into the primary multivariable model. In addition, dosage and adherence were not assessed. The binary exposure variable therefore does not distinguish between specific strains or formulations, indications, doses, or durations of use.

## 5. Conclusions

This study provides novel epidemiological insights into AD and probiotic use among young children in Serbia. Cesarean delivery, post-term birth, and probiotic use were independently associated with higher odds of parent-reported AD, although the cross-sectional design precludes causal interpretation, particularly regarding probiotic use. Probiotic use was highly prevalent among children with AD, but was predominantly related to antibiotic therapy and gastrointestinal complaints rather than to the prevention or reduction of skin symptoms. Parents of children with AD also showed somewhat more favorable attitudes toward probiotics, particularly regarding their potential role in preventing AD, although the magnitude of this difference was small. These findings suggest an opportunity for better-informed parental decision-making and evidence-based guidance from healthcare professionals regarding the potential role of probiotics in pediatric AD. Such guidance should take into account the increasing evidence suggesting that probiotic effects may be strain-specific and may depend on the duration of use, rather than assuming that all probiotic products are equivalent. Further prospective studies are needed to determine whether specific probiotic strains, formulations, and treatment durations may have a clinically relevant role in the prevention or management of pediatric AD.

## Figures and Tables

**Table 1:** Characteristics of the study sample (N = 1153).

Variable	Category	*n*	%
Atopic dermatitis	No	881	76.4
	Yes	272	23.6
Child Sex	Male	539	46.7
	Female	614	53.3
Child Age	1	314	27.2
	2	310	26.9
	3	273	23.7
	4	256	22.2
Mode of delivery	Cesarean section	437	37.9
	Vaginal delivery	716	62.1
Gestational age at birth	Full-term	1015	88.0
	Post-term	81	7.0
	Preterm	57	4.9
Infant feeding	Exclusive breastfeeding	639	55.4
	Mixed feeding	398	34.5
	Exclusive formula feeding	116	10.1
Number of children in the family	One	603	52.3
	Two	458	39.7
	Three or more	92	8.0
Probiotic use (in previous 12 months)	No	406	35.2
	Yes	747	64.8

**Table 2:** Association between demographic and early-life characteristics and the presence of AD among children aged 1–4 years.

Variable	Category	No AD, *n*	No AD, %	AD, *n*	AD, %	χ^2^ (df)	*p*-Value
Sex	Male	410	76.1	129	23.9	0.066 (1)	0.797
	Female	471	76.7	143	23.3		
Child age (years)	1	234	74.5	80	25.5	2.572 (3)	0.462
	2	234	75.5	76	24.5		
	3	218	79.9	55	20.1		
	4	195	76.2	61	23.8		
Mode of delivery	Cesarean section	312	71.4	125	28.6	9.813 (1)	**0.002**
	Vaginal delivery	569	79.5	147	20.5		
Gestational age at birth	Full-term	785	77.3	230	22.7	5.932 (2)	0.052
	Post-term	53	65.4	28	34.6		
	Preterm	43	75.4	14	24.6		
Infant feeding	Breastfeeding	488	76.4	151	23.6	1.741 (2)	0.419
	Mixed feeding	299	75.1	99	24.9		
	Formula feeding	94	81.0	22	19.0		
Number of children in the family	One	447	74.1	156	25.9	4.064 (2)	0.131
	Two	359	78.4	99	21.6		
	Three or more	75	81.5	17	18.5		
Probiotic use	No	333	82.0	73	18.0	10.943 (1)	**0.001**
	Yes	548	73.4	199	26.6		

Categorical variables are presented as number (*n*) and percentage (%). Pearson’s χ^2^ test was used to compare categorical variables between children with and without AD (AD vs. No AD). Statistically significant *p*-values are shown in bold.

**Table 3:** Multivariable logistic regression analysis of factors associated with AD among children aged 1–4 years.

Variable	aOR (Exp(B))	95% CI	*p*-Value
Sex (male vs. female)	1.071	0.812–1.413	0.628
Age (overall)	-	-	0.484
1 year vs. 4 years	1.014	0.687–1.496	0.946
2 years vs. 4 years	0.976	0.659–1.446	0.904
3 years vs. 4 years	0.762	0.501–1.159	0.204
Mode of delivery (Cesarean section vs. vaginal delivery)	1.562	1.179–2.069	**0.002**
Gestational age at birth (overall)	-	-	0.046
Preterm vs. full-term	1.003	0.531–1.892	0.993
Post-term vs. full-term	1.862	1.139–3.044	**0.013**
Infant feeding type (overall)	—	—	0.511
Mixed feeding vs. breastfeeding	1.034	0.767–1.394	0.826
Formula feeding vs. breastfeeding	0.762	0.458–1.267	0.294
Probiotic use (yes vs. no)	1.645	1.214–2.231	**0.001**

Abbreviations: aOR, adjusted odds ratio; CI, confidence interval. Reference categories: female sex, age 4 years, vaginal delivery, full-term birth, exclusive breastfeeding, and no probiotic use. Statistically significant *p*-values are shown in bold.

**Table 4:** Patterns of probiotic use among children with atopic dermatitis (*n* = 272).

Variable	Category	*n*	%
Ever received probiotics ^a^	Yes	265	97.4
	No	7	2.6
Reasons for probiotic use ^b,c^	During antibiotic therapy	184	69.4
	Gastrointestinal complaints	173	65.3
	Respiratory problems	18	6.8
	Prevention and strengthening of immunity	116	43.8
	Prevention and treatment of allergic reactions	56	21.1
	Prevention or reduction of skin symptoms	95	35.8
	Daily use	4	1.5
	Travel	2	0.8
Longest duration of use ^b^	Less than 10 days	63	23.8
	10–30 days	58	21.9
	1–3 months	57	21.5
	4–6 months	19	7.2
	More than 6 months	68	25.7
Reported probiotic composition ^b^	*Lactobacillus*	91	34.3
	*Bifidobacterium*	5	1.9
	*Saccharomyces*	25	9.4
	Combination of strains	70	26.4
	Composition not considered	74	27.9
	Other	0	0.0
Factors influencing probiotic selection ^b,c^	Physician recommendation	166	62.6
	Pharmacist recommendation	103	38.9
	Probiotic composition	92	34.7
	Price	8	3.0
	Brand	16	6.0
	Other	2	0.8

^a^ Percentages were calculated among all children with atopic dermatitis (*n* = 272). ^b^ Percentages were calculated among children who had ever received probiotics (*n* = 265). Seven children who had never received probiotics were excluded because the follow-up questions were not applicable. ^c^ Multiple responses were allowed; therefore, percentages may exceed 100%.

## Data Availability

The original contributions presented in this study are included in the article. Further inquiries can be directed to the corresponding author.
